# Increased activity in frontal motor cortex compensates impaired speech perception in older adults

**DOI:** 10.1038/ncomms12241

**Published:** 2016-08-02

**Authors:** Yi Du, Bradley R. Buchsbaum, Cheryl L. Grady, Claude Alain

**Affiliations:** 1 Rotman Research Institute, Baycrest Centre for Geriatric Care, Toronto, Ontario, Canada M6A 2E1; 2 Institute of Psychology, Chinese Academy of Sciences, Beijing 100101, China; 3 Department of Psychology, University of Toronto, Ontario, Canada M8V 2S4

## Abstract

Understanding speech in noisy environments is challenging, especially for seniors. Although evidence suggests that older adults increasingly recruit prefrontal cortices to offset reduced periphery and central auditory processing, the brain mechanisms underlying such compensation remain elusive. Here we show that relative to young adults, older adults show higher activation of frontal speech motor areas as measured by functional MRI during a syllable identification task at varying signal-to-noise ratios. This increased activity correlates with improved speech discrimination performance in older adults. Multivoxel pattern classification reveals that despite an overall phoneme dedifferentiation, older adults show greater specificity of phoneme representations in frontal articulatory regions than auditory regions. Moreover, older adults with stronger frontal activity have higher phoneme specificity in frontal and auditory regions. Thus, preserved phoneme specificity and upregulation of activity in speech motor regions provide a means of compensation in older adults for decoding impoverished speech representations in adverse listening conditions.

## Abstract (web summary)

Seniors have difficulties understanding speech in noisy environments and it is known that their brains compensate reduced sensory processing in auditory cortex by engaging prefrontal areas. Here the authors find that phoneme specificity and increased activity in speech motor regions provide a means of compensation for decoding impoverished speech in older adults.

## Graphical abstract

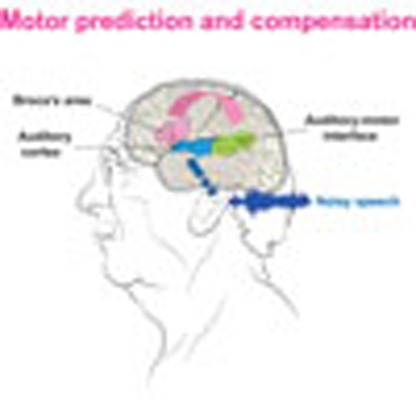

Perception and comprehension of spoken language—which involve mapping of acoustic signals with complex and dynamic structure to lexical representations (sound to meaning)—deteriorate with age^1^,^2^. Age-related decline in speech perception is further exacerbated in noisy environments, for example, when there is background noise or when several people are talking at once^3^,^4^. Prior neuroimaging research has revealed increased activity in prefrontal regions associated with cognitive control, attention and working memory when older adults processed speech under challenging circumstances^5^,^6^,^7^,^8^. These increased activations are thought to reflect a compensatory strategy of aging brains in recruiting more general cognitive areas to counteract declines in sensory processing^9^,^10^. However, a more precise accounting of the neural mechanism of such an age-related compensatory functional reorganization during speech perception in adverse listening conditions is lacking.

According to sensorimotor integration theories of speech perception^11^,^12^,^13^, predictions from the frontal articulatory network (that is, speech motor system), including Broca's area in the posterior inferior frontal gyrus (IFG) and ventral premotor cortex (PMv), provide phonological constraints to auditory representations in sensorimotor interface areas, for example, the Spt (Sylvian-parietal-temporal) in the posterior planum temporale (PT). This kind of sensorimotor integration is thought to facilitate speech perception, especially in adverse listening environments. In a recent functional magnetic resonance imaging (fMRI) study in young adults, we found greater specificity of phoneme representations, as measured by multivoxel pattern analysis (MVPA), in left PMv and Broca's area than in bilateral auditory cortices during syllable identification with high background noise^14^. This finding suggests that phoneme specificity in frontal articulatory regions may provide a means to compensate for impoverished auditory representations through top-down sensorimotor integration. However, whether older adults show preserved sensorimotor integration, and by which means they can benefit from it in understanding speech, particularly under noise-masking, has never been explicitly investigated.

In the current study, we measured blood oxygenation level-dependent (BOLD) brain activity while 16 young and 16 older adults identified naturally produced English phoneme tokens (/ba/, /ma/, /da/ and /ta/) either alone or embedded in broadband noise at multiple signal-to-noise ratios (SNR, −12, −9, −6, −2 and 8 dB). We find that older adults show stronger activity in frontal speech motor regions than young adults. These increased activations coincide with age-equivalent performance and positively correlate with performance in older adults, suggesting that the age-related upregulations are compensatory. We also assessed how well speech representations could be decoded in older brains using MVPA, which can detect fine-scale spatial patterns instead of mean levels of neural activity elicited by different phonemes. Older adults show less distinctive phoneme representations, known as neural dedifferentiation^15^,^16^,^17^,^18^,^19^, compared with young adults in speech-relevant regions, but the phoneme specificity in frontal articulatory regions is more tolerant to the degradative effects of both aging and noise than auditory cortices. In addition, older adults show a preserved sensorimotor integration function but deploy sensorimotor compensation at lower task demands (that is, lower noise) than young adults. To further probe the nature of age-related frontal upregulation in terms of its relationship with phoneme representations in speech-relevant regions, we tested whether under noise-masking activity in frontal articulatory regions would correlate with phoneme specificity in frontal and auditory regions in older adults. We show that older adults with stronger frontal activity have higher phoneme specificity, which indicates that frontal speech motor upregulation specifically improves phoneme representations. These results provide neural evidence that in older adults increased recruitment of frontal speech motor regions along with maintained specificity of speech motor representations compensate for declined auditory representations of speech in noisy listening circumstances.

## Results

### Behaviours

**Figure 1:**
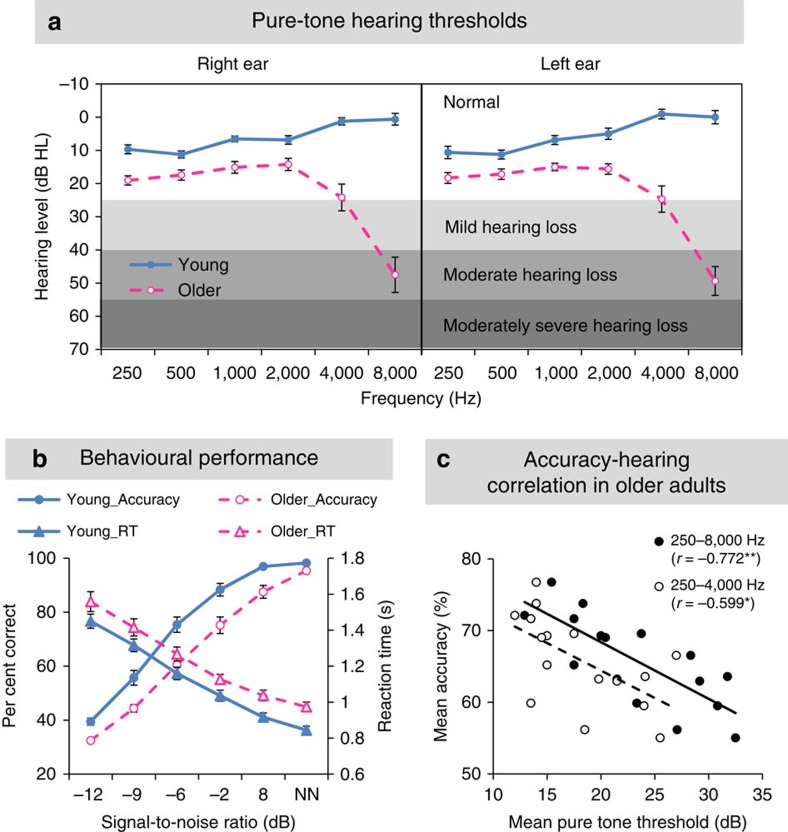
Hearing level and behavioural performance. (**a**) Group mean pure-tone hearing thresholds at each frequency for young and older adults. Error bars indicate s.e.m. (**b**) Group mean accuracy (left axis) and reaction time (right axis) across syllables as a function of SNR in both groups. NN represents the NoNoise condition. Error bars indicate s.e.m. (**c**) Correlations between the mean accuracy across syllables and SNRs and the mean pure-tone threshold across frequencies from 250 to 4,000 Hz (triangles) or from 250 to 8,000 Hz (circles) in older adults. *P<0.05; **P<0.01 by Pearson's correlations.

All participants had normal (<25 dB HL^20^) pure-tone threshold at both ears from 250 to 4,000 Hz, the frequency range relevant for speech perception^21^, except for six older adults who had mild-to-moderate hearing loss at 4,000 Hz (Fig. 1a). All older adults had some hearing loss at 8,000 Hz. A mixed-effects analysis of variance (ANOVA) showed that older adults had higher ear-mean hearing threshold than young adults at all frequencies (*F*_1,30_=94.47, *P*<0.001), with more severe hearing loss at higher (4,000 and 8,000 Hz) frequencies (group × frequency: *F*_5,150_=38.2, *P*<0.001).

Participants' accuracy and reaction time did not differ by syllable in either group, so the mean accuracy and reaction time across syllables are used hereafter. A 6 (SNR) × 2 (group) mixed ANOVA on arcsine-transformed^22^ accuracy revealed that older adults were less accurate than young adults irrespective of SNR (*F*_1,30_=19.48, *P*<0.001), and accuracy increased with increasing SNR in both groups (*F*_5,150_=399.10, *P*<0.001), with a marginally significant group × SNR interaction (*F*_5,150_=2.21, *P*=0.056, Fig. 1b). Older adults responded more slowly than young adults regardless of SNR (*F*_1,30_=6.61, *P*=0.015), and reaction time decreased with elevating SNR in both groups (*F*_5,150_=244.86, *P*<0.001), with no group × SNR interaction (*F*_5,150_=0.24, *P*=0.95).

Notably, in older adults the overall accuracy across syllables and SNRs negatively correlated with the mean pure-tone thresholds both at speech-relevant frequencies (250 to 4,000 Hz, *r*=−0.599, *P*=0.014), and across all frequencies including 8,000 Hz, which was most affected by aging (*r*=−0.772, *P*<0.001; Fig. 1c). However, in older adults neither did the overall accuracy correlate with age (*r*=0.244, *P*=0.36) nor did age correlate with the mean hearing level across either frequency range (both *r*<0.39, *P*>0.13). Thus, peripheral hearing loss partially contributed to impaired speech in noise perception in older adults.

### Age-related frontal upregulation is compensatory

**Figure 2:**
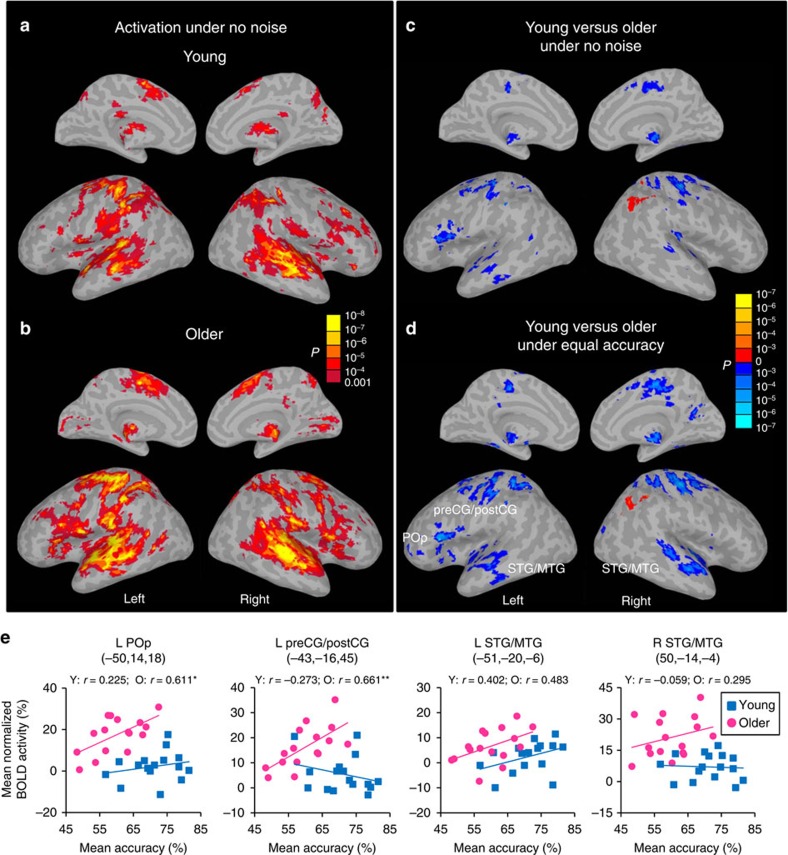
Age difference in BOLD activity. Activity elicited by syllable identification at the NoNoise condition in young (**a**) and older adults (**b**). Activity in young adults versus activity in older adults at the NoNoise condition (**c**) and conditions when two groups equalled in accuracy (average activity at −6 and −2 dB SNRs in young versus average activity at −2 and 8 dB SNRs in older) (**d**). Results are thresholded at *P*FWE<0.01. (**e**) Correlations between the mean activity across −12 to 8 dB SNRs in four ROIs (left POp, left preCG/postCG and bilateral STG/MTG) and the mean accuracy across those SNRs in older (red circles) and young adults (blue squares). The coordinates are in Talairach space. *P<0.05; **P<0.01 by Pearson's correlations. POp, pars opercularis; preCG/postCG, precentral and postcentral gyrus; STG/MTG, superior and middle temporal gyrus.

Compared with the inter-trial baseline, identification of syllables presented without noise (NoNoise condition) activated bilateral superior and middle temporal regions, bilateral inferior, middle and medial frontal regions, bilateral inferior and superior parietal regions, the thalamus, as well as the left dorsal motor and somatosensory regions in young adults (Fig. 2a, family-wise error-corrected *P*-value (*P*FWE)<0.01). Older adults showed similar activation patterns but with larger amplitude, especially in left frontal and bilateral temporal, motor and somatosensory regions (Fig. 2b). A group contrast of BOLD activity at the NoNoise condition (Fig. 2c, *P*FWE<0.01) and conditions with matched accuracy (the mean activity at −6 and −2 dB SNRs in young versus the mean activity at −2 and 8 dB SNRs in older adults, Fig. 2d and Table 1, *P*FWE<0.01), revealed similar age-related changes. That is, compared with young adults, older adults showed higher activity in the left pars opercularis (POp) of Broca's area (BA44) and adjacent PMv (BA6), and bilateral regions in the anterior and middle superior temporal gyrus (STG) and middle temporal gyrus (MTG), dorsal precentral gyrus (preCG) (including both motor and premotor cortices) and postcentral gyrus (postCG), superior parietal lobule, medial frontal gyrus and thalamus; but lower activity in the right inferior parietal lobule. Thus, increased activity in older listeners was associated with an age-equivalent performance.

We further assessed whether upregulation of activity in frontal or auditory regions in older adults benefited behavioural performance across participants in noise masking conditions. Four spherical (8-mm radius) regions-of-interest (ROIs) were centred at the peak voxels that showed significant age differences under matched accuracy: left POp (−50, 14, 18), left preCG/postCG (−43, −16, 45), left STG/MTG (−51, −20, −6) and right STG/MTG (50, −14, −4). The brain–behaviour correlations were carried out between the mean activity in each of the four ROIs and the mean accuracy across all the SNRs (that is, −12, −9, −6, −2 and 8 dB). For older adults, the mean activity across −12 to 8 dB SNRs in the left POp (*r*=0.611, *P*=0.012, false-discovery rate (FDR)-corrected *P*<0.05) and left preCG/postCG (*r*=0.661, *P*=0.005, FDR-corrected *P*<0.05) positively correlated with the mean behavioural accuracy across those SNRs (Fig. 2e). Such a correlation was not found in the left STG/MTG (*r*=0.483, *P*=0.058) and right STG/MTG (*r*=0.295, *P*=0.268). After controlling for the mean pure-tone threshold at speech-relevant frequencies, activity in the left POp and preCG/postCG showed a trend of correlation with accuracy in older adults (partial *r*=0.604 and 0.612, uncorrected *P*=0.017 and 0.015, respectively, FDR-corrected *P*>0.05). However, none of the correlations were significant in young adults (all |*r*|<0.41, *P*>0.12), and the correlation coefficient significantly differed between groups in the left preCG/postCG (*Z*=2.74, *P*=0.006, FDR-corrected *P*<0.05), but not in other ROIs (*z*<−1.23, *P*>0.21). Thus, stronger activity in speech motor areas (that is, left POp and premotor cortex) was associated with better performance under noise masking in older listeners, consistent with an aging-related compensatory upregulation of frontal regions during speech in noise perception.

### Age-related phoneme dedifferentiation

**Figure 3:**
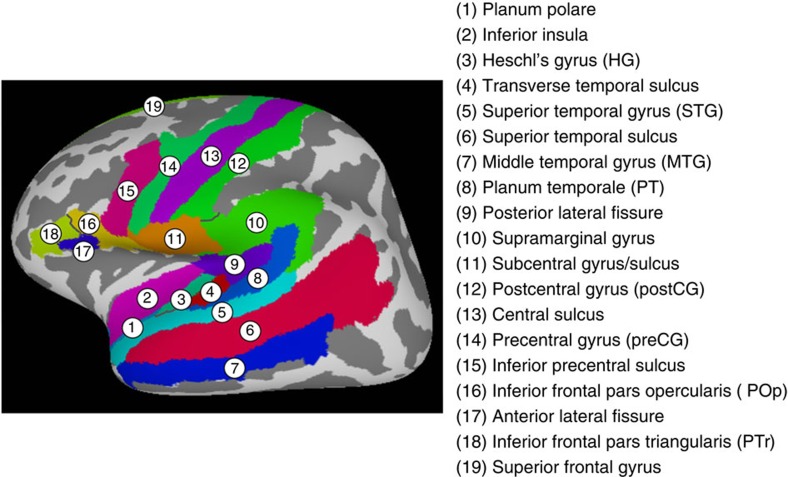
Speech-relevant anatomical ROIs used in multivoxel pattern analysis. The ROI mask consisting of 19 left and 19 right ROIs was created by intersecting a Neurosynth automated meta-analysis (search term: ‘speech') and the 148 Freesurfer anatomical ROIs (aparc 2009 atlas).

**Figure 4:**
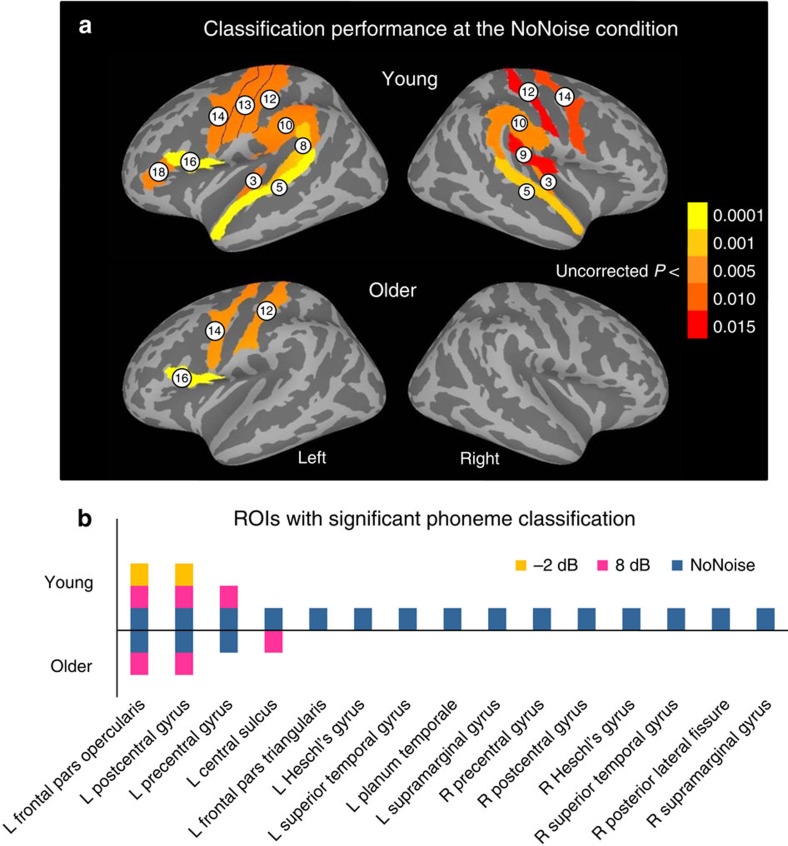
Phoneme classification performance. (**a**) Regions with significant phoneme classification (AUC>0.5, one-sample *t*-test with FDR-corrected *P*<0.05) at the NoNoise condition for each group. (**b**) Overview of ROIs and SNRs when significant classification was revealed in each group. SNRs at which each ROI showed significant classification are displayed on the *y*-axis, with ROI labels listed across the *x*-axis. The top half shows data from young adults and the bottom half shows data from older adults. 3, Heschl's gyrus; 5, superior temporal gyrus; 8, planum temporale; 9, posterior lateral fissure; 10, supramarginal gyrus; 12, postcentral gyrus; 13, central sulcus; 14, precentral gyrus; 16, pars opercularis; 18, pars triangularis.

MVPA was performed within 38 anatomical ROIs in both hemispheres (Fig. 3) that are important for speech perception and production, as determined by a coordinate-based meta-analysis (see the ‘Methods' section). Multivariate classifiers were trained to discriminate activity patterns associated with different phonemes using shrinkage discriminant analysis^23^ and then tested on independent sets of trials using five-fold cross-validation. When young adults identified syllables presented without noise, significant phoneme classification (area under the curve (AUC)>0.5 chance level, one-sample *t*-tests with FDR-corrected *P*<0.05) was observed in bilateral regions in auditory cortex including Heschl's gyrus (HG) and STG, supramarginal gyrus, postCG and preCG, as well as the left PT and Broca's area including both the POp and pars triangularis (Fig. 4a,b and Table 2). For older adults, even when noise was absent, phoneme representations could not be reliably distinguished in bilateral temporal-parietal regions, with significant classification found only in the left postCG, preCG and POp (Fig. 4a,b and Table 2). The comparison of classification performance between the two groups at the NoNoise condition suggests an age-related dedifferentiation of speech representations, which was more severe in lower-level (for example, auditory cortex) relative to higher-level (for example, prefrontal cortex) cortical regions of the speech processing hierarchy.

Age-related phoneme dedifferentiation was also reflected as equivalent phoneme classification performance at the NoNoise condition in older adults and at 8 dB SNR in young adults, where the left POp, preCG and postCG showed significant phoneme specificity in both cases (Fig. 4b and Table 2). Not surprisingly, the two groups showed similar behavioural accuracy under such a comparison (Fig. 1b). This indicates that aging appeared to add noise to speech representations and behaviours accordingly.

In addition, a mixed ANOVA with ROI as the within-subject factor, group as the between-subject factor and SNR as the covariate on AUC scores revealed a marginally significant reduction in phoneme classification in older compared with young adults regardless of ROI and SNR (*F*_1,189_=2.993, *P*=0.085).

### Greater phoneme specificity in frontal than auditory regions

Intriguingly, brain regions differed in the robustness of phoneme specificity against noise masking in a way that was not substantially affected by aging. For young adults, distinctive phoneme representations were not detected in bilateral temporal and parietal regions, but maintained in the left postCG and frontal articulatory regions (that is, preCG and POp) at the 8 dB SNR (Fig. 4b top half and Table 2). Phoneme classification was significant in the left postCG and POp even at −2 dB but not lower SNRs in young adults. Although aging was associated with phoneme dedifferentiation in speech-relevant regions, phoneme specificity was better preserved in frontal articulatory regions than in auditory cortices, and this advantage was not affected by relatively weak noise masking. That is, in older adults classification was significant in the left POp when SNR≥8 dB, whereas no reliable classification was found in auditory regions even without noise (Fig. 4b bottom half and Table 2). When SNR<8 dB, although older listeners identified syllables above the chance level, no ROI showed significant classification.

### Shifted sensorimotor integration function in older adults

**Figure 5:**
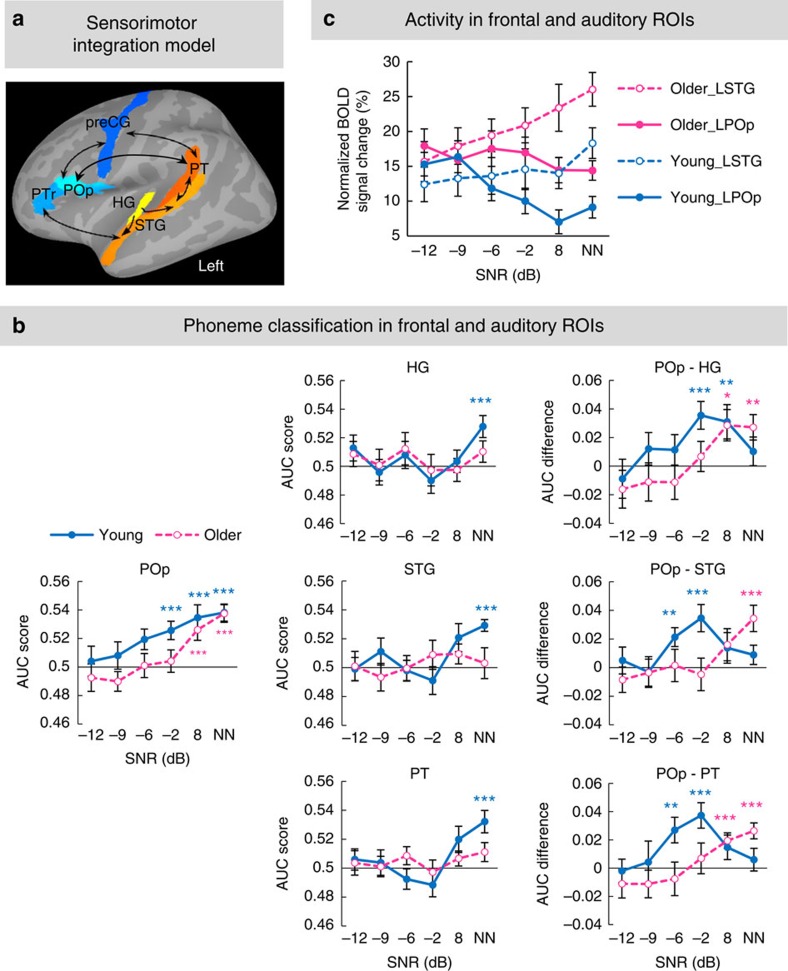
Phoneme classification performance and BOLD activity in frontal and auditory ROIs. (**a**) Six anatomical ROIs in the left hemisphere (3 frontal ROIs: POp, PTr and preCG; 3 auditory ROIs: HG, STG and PT) composing the sensorimotor mapping model are displayed on an inflated surface. The model was modified from Fig. 5 in Rauschecker and Scott^13^. (**b**) The AUC scores as a function of SNR in the left POp (left panel) and each auditory ROI (HG, STG and PT, middle panel) are shown for each group. ***P<0.005 by one-sample *t*-tests showing conditions with significant classification in young (blue asterisks) or older (red asterisks) adults. Differences in the AUC score between the left POp and each auditory ROI as a function of SNR are shown for each group in the right panel. *P<0.05; **P<0.01; ***P<0.005 by one-sample *t*-tests indicating SNRs at which classification in the POp was stronger than in one of auditory ROIs in young (blue asterisks) or older (red asterisks) adults. Notably, sensorimotor integration between frontal and auditory regions as a function of SNR was right shifted in older adults. (**c**) BOLD activity as a function of SNR in the left POp (solid lines) and left STG (dash lines) anatomical ROIs in young (blue lines) and older (red lines) adults.

Regardless of age, the greater phoneme specificity observed in frontal articulatory regions than in auditory and sensorimotor interface regions appears to be a neural marker of top-down sensorimotor mapping during speech in noise perception. To examine how the sensorimotor integration function varied with task demands (that is, SNR) and age, the AUC scores were compared between the frontal POp and three auditory ROIs (HG, STG and PT) in the left hemisphere, the core regions in the proposed sensorimotor integration model (Fig. 5a). Generally, phoneme specificity was revealed under stronger noise masking in the left POp than in auditory ROIs regardless of age (Fig. 5b, left and middle panels). Specificity linearly increased with elevating SNR in the left POp and non-linearly changed with SNR in auditory ROIs in young adults, but not in older adults. Moreover, phoneme classification was stronger in young than older adults at medium-to-high SNRs depending on ROI.

For each frontal-auditory pairwise ROIs, a 2 (ROI) × 6 (SNR) × 2 (group) mixed ANOVA revealed a significant ROI × SNR interaction between the POp and HG (*F*_5,150_=3.94, *P*=0.002), the POp and STG (*F*_5,150_=2.60, *P*=0.027), and the POp and PT (*F*_5,150_=2.68, *P*=0.024). The AUC difference scores were then calculated by subtracting each auditory ROI value from the POp value at each SNR (Fig. 5b, right panel), and put into a 6 (SNR) × 2 (group) mixed ANOVA to assess whether the classification difference between the POp and each auditory ROI as a function of SNR differed with age. None of the pairwise AUC difference scores showed a significant SNR × group interaction (all *F*_5,150_<1.9, *P*>0.1). However, the AUC difference scores significantly changed with SNR in each group (all *F*_5,75_>2.51, *P*<0.038, repeated-measures ANOVAs) with a quadratic trend in young adults (all *F*_1,15_>4.63, *P*<0.048) and a linear trend in older adults (all *F*_1,15_>7.72, *P*<0.015). Follow-up one-sample *t*-tests revealed significantly stronger classification in the POp than in each auditory ROI at medium to low noise levels (−6 dB≤SNR≤8 dB) in young adults (all *t*_15_>2.93, *P*<0.011). In contrast, for older adults stronger classification in the POp compared with auditory ROIs was only observed in low or no noise conditions (SNR≥8 dB; all *t*_15_>2.57, *P*<0.021, see Fig. 5b right panel for details).

These results suggest a convex pattern of sensorimotor mapping from frontal speech motor areas to auditory regions in young adults as a function of noise, which peaked at a medium SNR level (−6 to −2 dB). In comparison, sensorimotor integration function was preserved but shifted to easier task levels (SNR≥8 dB, the curve was shifted to the right) in the older group, indicating an increased need for speech motor modulation in aiding speech perception in older adults.

### Phoneme specificity is independent of overall BOLD activity

Importantly, the inter-regional or group difference in the specificity of phoneme representations was independent from the regional or group difference in the mean BOLD activity. As shown in Fig. 5b, phoneme specificity was greater in frontal articulatory regions (for example, left POp) than in auditory areas (for example, left STG) in both groups, and greater in young adults compared with older adults in both frontal and auditory regions. To examine whether higher phoneme specificity arose from stronger BOLD activation in one compared with the other region or group, the mean BOLD activities in the anatomically defined left POp and left STG were subjected to a 2 (ROI) × 6 (SNR) × 2 (group) mixed ANOVA. This revealed significant main effects of ROI (*F*_1,30_=6.38, *P*=0.017) and group (*F*_1,30_=4.32, *P*=0.046), and significant ROI × SNR (*F*_5,150_=33.61, *P*<0.001) and group × SNR (*F*_5,150_=3.12, *P*=0.01) interactions (Fig. 5c). For the ROI × SNR interaction, paired-samples *t*-tests revealed significantly lower activity in the left POp than left STG when SNR≥8 dB in both groups (all *t*_15_>3.29, *P*≤0.005). For the group × SNR interaction, independent-samples *t*-tests revealed higher activity in older than young adults in the left POp when SNR≥−2 dB (all *t*_30_>2.41, *P*<0.025) and in the left STG when SNR≥8 dB (both *t*_30_>2.32, *P*<0.03). Thus, neither the reduced phoneme specificity in auditory compared with frontal articulatory regions, nor the decrease in phoneme specificity in older compared with young adults, was associated with decreased mean BOLD activity in auditory regions or the older group, respectively. In addition, older adults exhibited consistently elevated activation in the left POp with a reduced dynamic range in response to changing task demands, unlike young adults, who showed a steep increment of left POp activity with increasing noise (Fig. 5c).

### Frontal upregulation compensates for phoneme specificity

**Figure 6:**
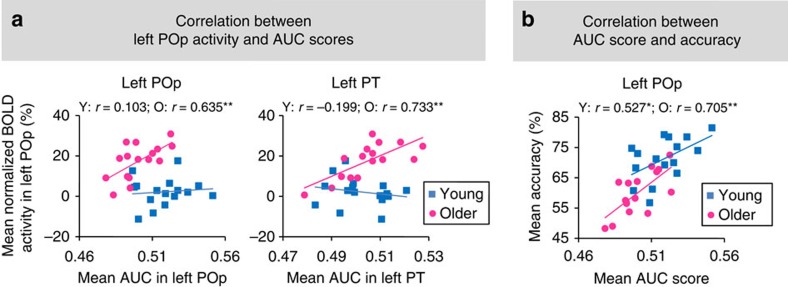
Relationships between frontal activity and phoneme specificity and behavioral accuracy. (**a**) The mean activity across −12 to 8 dB SNRs in the POp spherical ROI (−50, 14, 18; 8-mm radius) positively correlated with the mean AUC score across those SNRs in the left POp and left PT in older adults (open circles) but not in young adults (filled squares). (**b**) The mean AUC score across −12 to 8 dB SNRs in the left POp positively correlated with the mean accuracy at those SNRs in both age groups. *P<0.05; **P<0.01 by Pearson's correlations.

We further assessed the relationships between frontal activity at the mean hemodynamic response level, phoneme specificity at the representational level and accuracy at the behavioural level to understand the nature of age-related frontal upregulation. The increased activity in speech motor regions could reflect compensation that directly improved phoneme specificity in speech-relevant regions via sensorimotor integration, or a more general increased demand on cognitive processes, such as attention, verbal working memory and categorical judgment. To distinguish between these two possibilities, we investigated the relationships between the mean activity at noise masking conditions (−12 to 8 dB SNRs) in the left POp spherical ROI (the same ROI in Fig. 2e) and average phoneme specificity across those SNRs in four core regions (the left POp, HG, STG and PT) of the sensorimotor integration network. The mean activity in the left POp positively correlated with the mean AUC score in the left POp (*r*=0.635, *P*=0.008, FDR-corrected *P*<0.05) and left PT (*r*=0.733, *P*=0.001, FDR-corrected *P*<0.05) in older adults, but not in young adults (both |*r*|<0.2, *P*>0.45, Fig. 6a). Such a correlation was not found for the left HG or STG in either group (all *r*<0.34, *P*>0.2). Furthermore, the correlation coefficient significantly differed between groups in the left PT (*z*=2.9, *P*=0.002, FDR-corrected *P*<0.05) but not in other ROIs (*z*<1.7, *P*>0.09). Therefore, under noise masking older adults with stronger left POp activity had greater phoneme specificity in that region as well as in the left PT.

We then tested whether phoneme specificity in the left POp or auditory ROIs (HG, STG and PT) correlated with participant's accuracy in syllable identification. The mean AUC score across −12 to 8 dB SNRs in the left POp positively correlated with the mean behavioural accuracy across those SNRs in older adults (*r*=0.705, *P*=0.002, FDR-corrected *P*<0.05) but not in young adults (*r*=0.527, *P*=0.036, FDR-corrected *P*>0.05), without a significant group difference in the correlation coefficient (*z*=0.74, *P*=0.459) (Fig. 6b). The brain–behaviour correlation at noise masking conditions was not significant in the left HG, STG or PT in either group (all|*r*|<0.39, *P*>0.14). We propose that positive relationships among frontal upregulation, phoneme specificity in the left POp and behavioural accuracy in older adults supports a specific compensatory mechanism in which the recruitment of speech motor areas provides a means to facilitate speech identification, particularly at noisy conditions, by boosting the specificity of speech representations.

## Discussion

The present study revealed increased activity in speech motor regions of older adults compared with young adults, which remained after controlling for the age difference in performing syllable identification in noise. Importantly, under noise masking the positive correlation between activity in speech motor regions and behavioural accuracy in older adults is consistent with a compensatory frontal upregulation. Using MVPA, we found greater phoneme specificity in frontal articulatory regions than in auditory areas despite a general dedifferentiation of phoneme representations in older adults. Furthermore, the sensorimotor integration function, reflected in greater phoneme specificity in frontal motor than auditory regions, was shifted towards lower task demands in older adults compared with young adults, suggesting that older adults relied to a greater extent on speech motor compensation during speech in noise perception relative to younger counterparts. Notably, the lower phoneme specificity in auditory compared with frontal regions at medium-to-no noise conditions in both groups was not due to reductions in BOLD activity in auditory regions. Also, the reduced phoneme specificity in older compared with young adults in both frontal and auditory regions cannot be accounted for by decreased BOLD activation in older adults. Under noise masking increasing activity in left POp correlated with higher phoneme specificity in this region and in the sensorimotor interface in left PT in older adults, which functionally linked frontal speech motor upregulation with disambiguation of phonological representations via sensorimotor integration. Taken together, these findings provide neuroimaging evidence for a preserved sensorimotor integration function during perception of speech in noise in seniors, and for the first time, integrate the decline-compensation hypothesis^9^,^10^ with age-related dedifferentiation^24^. That is, older adults enhanced frontal speech motor recruitment to compensate for auditory dedifferentiation during speech comprehension in noisy environments.

In older adults the pure-tone threshold correlated with overall accuracy in syllable identification. Therefore, a reduction in peripheral hearing acuity partly contributed to impaired performance for older adults in the current study, although it alone cannot adequately account for speech in noise perception deficits^3^,^25^. Consistent with previous findings that showed age-related upregulation of activity in various cognitive tasks^26^ and speech perception in particular^5^,^6^,^7^,^8^, we found increased activities in multiple temporal, frontal and parietal regions in older relative to young adults. Importantly, upregulation of left POp of Broca's area and motor/premotor regions in preCG accompanied age-equivalent performance, and the activity in those regions positively correlated with older adults' ability to correctly identify speech in noise. Our results fit with the compensatory account^9^,^10^,^27^ that the additional activity in speech motor regions presented a beneficial function in supporting task performance. In line with previous studies in young adults that frontal articulatory regions were activated in perceiving speech even without any overt speech production task^14^,^28^,^29^,^30^, increased recruitment of speech motor regions in older adults could reflect elevated motoric feedback modulation on speech perception in counteracting deficient auditory processing. The frontal speech motor upregulation may result from adaptive changes in resource allocation and/or coping strategy from data-driven sensory processing towards experience/knowledge-based top-down predictions from articulatory gestures.

Aging is associated with dedifferentiated neural processes in sensory, memory and motor systems^15^,^16^,^17^,^18^,^19^ and a reduction in the distinctiveness of neural representations may serve as a common cause for general cognitive disruptions^24^,^26^. Here, we revealed an age-related dedifferentiation of phoneme representations in the cortical hierarchy of speech processing, reflected as reduced number of regions showing significant phoneme classification when there was no noise and comparable classification pattern at the NoNoise condition in older adults and at 8 dB SNR in young adults. The dedifferentiation in older listeners may be due to age-related declines in peripheral hearing^1^, deficits in phase-locking and timing in brainstem^31^,^32^, changes in cortical anatomy^33^,^34^, as well as reductions in functional connectivity^6^,^24^. Reduced phoneme specificity in speech-relevant regions may partially account for the difficulty in fine discrimination of syllables in the elderly. However, when there was noise, phoneme classification performance only in left POp, not in auditory cortices, correlated with syllable identification accuracy in older adults, reflecting the impact of articulatory predictions in driving categorical decisions in adverse listening conditions.

Intriguingly, the age-related phoneme dedifferentiation was not evenly distributed in speech-relevant regions. Phoneme specificity was better preserved in frontal articulatory regions than auditory areas in older adults, both in the NoNoise and slightly noisy conditions. Specifically, the left POp showed better phoneme classification than auditory areas at medium (−6 to 8 dB SNRs) but not lower or higher noise levels in young adults, indicating a convex pattern of sensorimotor mapping as a function of task difficulty. In comparison, older adults exhibited stronger phoneme classification in left POp than in auditory regions only when noise was weak or absent (SNR≥8 dB), leading to a preserved sensorimotor integration function which, however, was shifted to easier task levels. The stronger reliance on sensorimotor compensation by older listeners was also suggested by elevated left POp activity irrespective of task difficulty, in contrast to a SNR-dependent increase of left POp activity in young adults. The persistent upregulation with reduced dynamic responding range in speech motor regions along with the shifted sensorimotor integration function in older listeners are consistent with the compensation-related utilization of neural circuits hypothesis (CRUNCH^35^), such that older adults recruited sensorimotor integration at lower levels of task load than young adults.

However, we do not know yet whether frontal speech motor activity still affects older adults' performance when SNR falls below a certain level (for example, −2 dB here or even lower when scanner noise is absent). It would be important for future work to investigate the effects of deactivation of frontal articulatory regions via transcranial magnetic stimulation or other approaches on the speech in noise identification performance at various SNRs in older adults. Moreover, the constant scanner noise in the background might increase the cognitive load, especially in older adults, and affect the brain activity patterns in general. It is possible that higher phoneme specificity would be observed and older adults may show less need of speech motor compensation in ‘quiet' environments.

Note that neither the lower phoneme specificity in auditory relative to frontal articulatory regions in both groups, nor the decreased phoneme specificity in older compared with young adults in both frontal and auditory regions, was due to decreased hemodynamic responses. Instead, stronger BOLD activity was found in auditory than frontal regions regardless of age, and activity was stronger in older relative to young adults in both auditory and frontal regions. Our findings suggest that reduced phoneme distinctiveness in older adults and in auditory regions may be the result of ‘noisy' phonological representations associated with elevated activity strength, possibly caused by neural inefficiency, but less clear, consistent and differential patterns. The differential robustness of phoneme representations in frontal versus auditory regions to the effects of aging and noise masking may also arise from the hierarchical organization of speech processing from data-driven sensory processing in auditory cortices to schema-driven linguistic and decision processes in frontal motor regions^11^,^13^,^36^.

Since categorical speech perception requires listeners to maintain sub-lexical representations in an active state as a meta-linguistic judgment is made, increased activity in left IFG of older adults may reflect effort-related changes in attention^7^,^8^, verbal working memory^8^,^37^, cognitive control^5^ or categorical judgment and response selection^29^,^38^. However, the correlations among increased left POp activity, better phoneme classification in that region and in left PT, and improved performance in older adults under noise masking directly link frontal speech motor upregulation with a specific compensatory mechanism in enhancing the specificity of speech representations, which in turn facilitated speech in noise identification. The positive correlation between left POp's activity and phoneme specificity in left PT suggests that neural dedifferentiation in auditory regions may be a target of frontal compensation. That is, increased activity in frontal speech motor regions in older adults may counteract the lack of phoneme discrimination in auditory cortex, likely through top-down sensorimotor mapping.

In summary, we revealed an age-related increase of activity in speech motor regions that compensated for performance and dedifferentiated phoneme representations during speech in noise perception. We were able to show a link between compensatory frontal upregulation and neural dedifferentiation associated with aging in a sensorimotor integration framework. The relation between preserved phoneme specificity and the upregulation in frontal articulatory regions in seniors provides evidence that sensorimotor integration serves as a source/mechanism of compensation for speech perception in challenging listening conditions, which significantly advances our understanding of the age-related increase of frontal activity. The recruitment of the frontal speech motor system in understanding speech interacted with cognitive demands and age; seniors called on sensorimotor integration at easier task conditions than their younger counterparts. Our findings also suggest that phoneme dedifferentiation may be a neural correlate for difficulty with speech in noise perception, and the binding of bottom-up sensory processing with top-down articulatory predictions substantially impacts speech recognition performance in the elderly. Moreover, the preserved sensorimotor integration function in seniors suggests avenues for rehabilitative and training regimens for better communication later in life.

## Methods

### Participants

Sixteen young adults aged between 21 and 34 years old (M=26.2±4.7, 8 females) and 16 older adults aged between 65 and 75 years old (M=70.4±3.5, 9 females) participated in the study. All participants gave written informed consent. The study was approved by the University of Toronto and Baycrest Hospital Human Subject Review Committee. All participants were native English speakers and right-handed. Pure-tone hearing levels for both groups of participants are shown in Fig. 1a. Data from all participants entered analyses.

### Stimuli and task

The stimuli were four naturally produced English consonant-vowel syllables (/ba/, /ma/, /da/ and /ta/), spoken by a female talker (standardized UCLA version of the Nonsense Syllable Test^39^). Each syllable token was 500-ms in duration and matched in terms of average root-mean-square sound pressure level. The vowel was always /a/ (as in father) because its formant structure provides a superior SNR relative to the MRI scanner spectrum. The four phonemes were chosen for their balanced features on place of articulation (labial /b/ and /m/ versus alveolar /d/ and /t/). A 500-ms white-noise segment (4-kHz low-pass cutoff, 10-ms rise-decay envelope) starting and ending simultaneously with the syllables was used as the masker. Sounds were presented via circumaural MRI-compatible headphones (HP SI01, MR Confon, Magdeburg, Germany), acoustically padded to suppress scanner noise by 25 dB. The intensity level of syllables was fixed at 85 dB, the noise level was adjusted to 97, 94, 91, 87, 77 or 0 dB, leading to five levels of SNR (−12, −9, −6, −2 and 8 dB) and the NoNoise condition. SNR was thus inversely related to the overall sound level. The SNR levels were chosen on the basis of a pilot behavioural study with five young adults, which revealed a quasi-linear relationship with accuracy for all four syllables.

Before scanning, syllables were presented individually without noise (four trials per syllable), and participants identified the syllables by pressing one of four keys on a parallel four-button pad using their right hand fingers (index to little fingers in response to /ba/, /da/, /ma/ and /ta/ sequentially) with an accuracy of 94% or better. During scanning, 80 noise-masked syllables (four trials per syllable per SNR) and 20 syllables alone (five trials per syllable) were randomly presented in each block with an average inter-stimuli-interval of 4 s (2–6 s, 0.5 s step), and five blocks were given in total. Participants were asked to listen carefully and identify syllables as fast as possible by pressing corresponding keys on a parallel four-button pad using their right fingers as trained outside the scanner. No counterbalance on finger-syllable associations among participants was applied.

### Behavioural data analysis

Both the percentage of trials correctly identified and RT (using both correct and incorrect trials) were computed for each syllable at each noise condition. To exclude the influence of restricted range of the percent accuracy at 0 to 100%, the statistics was applied to the percent accuracy after arcsine transformation^22^


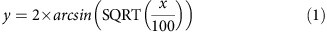


where, *y* is the arcsine transformed accuracy, *x* is the percent accuracy.

Arcsine-transformed accuracy and RT across syllables were then subjected to a mixed ANOVA with age as the between-subject factor and SNR as the within-subject factor separately. Older adults' overall accuracies and mean pure-tone thresholds were additionally subjected to a Pearson's correlation to reveal the relationship between peripheral hearing level and performance.

### MRI acquisition and data pre-processing

Participants were scanned using a Siemens Trio 3T magnet with a standard 12-channel ‘matrix' head coil. T2*-weighted functional images were collected with a continuous echo-planar imaging sequence (30 slices, matrix size=64 × 64, 5-mm thick, TR=2,000 ms, TE=30 ms, flip angle=70°, FOV=200 mm, voxel size=3.125 × 3.125 × 5 mm). High-resolution T1-weighted anatomical images were acquired after three functional runs using SPGR (axial orientation, 160 slices, 1-mm thick, TR=2,000 ms, TE=2.6 ms, FOV=256 mm).

The fMRI data were pre-processed using Analysis of Functional Neuroimages software (AFNI 2011 (ref. 40). In the pre-processing stage, fMRI data were spatially co-registered to correct for head motion using a 3D Fourier transform interpolation. For each run, images acquired at each point in the time-series were aligned volumetrically to a reference image acquired during the scanning session using the 3dvolreg plugin in AFNI. The pre-processed images were then concatenated and analysed by univariate General Linear Model (GLM) and MVPA.

### GLM analysis

Single-subject multiple-regression modelling was performed using the AFNI program 3dDeconvolve. Data were fit with different regressors for the four syllables and six SNRs. The predicted activation time course was modelled as a ‘gamma' function convolved with the canonical hemodynamic response function. For each noise level, the four syllables were grouped and contrasted against the baseline (silent inter-trial intervals), as the GLM revealed similar activity across syllables. Individual contrast maps were normalized to Talairach stereotaxic space, re-sampled (voxel size=3 × 3 × 3 mm), and spatially smoothed using a Gaussian filter (FWHM=6.0 mm).

Individual maps at each noise level were then subjected to separate mixed ANOVAs with age as the between-subject factor to test the random effects for each group as well as the age difference in BOLD activity at each SNR. Since the accuracy at −6 (75.4±2.8%) and −2 dB (88.4±2.3%) SNRs in young adults equalled the accuracy at −2 (75.2±3.1%) and 8 dB (87.6±2.4%) SNRs in older adults, respectively, the mean activity at −6 and −2 dB SNRs in young adults and the mean activity at −2 and 8 dB SNRs in older adults were subjected to an additional mixed ANOVA to reveal the age difference on BOLD activity under equal performance. To correct for multiple comparisons, a cluster spatial extent threshold was applied by using AlphaSim with 1000 Monte Carlo simulations and contrast-specific smoothness of residual errors. This procedure yielded a *P*FWE<0.01 by using an uncorrected *P*<0.001 and removing clusters<15 voxels for activity at the NoNoise condition in both groups. For group difference maps, this yielded a *P*FWE<0.01, with an uncorrected *P*<0.01, and cluster size≥16 voxels for the NoNoise condition and 35 voxels for the equal performance condition. To display statistics at the group level, the statistic of interest was projected onto a cortical inflated surface template using surface mapping with AFNI (SUMA).

Four 8-mm radius spherical ROIs in the left POP (−50, 14, 18), left preCG/postCG (−43, −16, 45), left STG/MTG (−51, −20, −6) and right STG/MTG (50, −14, −4) were centred at the peak voxels as showing significant age difference in activity under equal performance (*P*FWE<0.01). The preCG/postCG ROI occupied a part of both areas, so as the STG/MTG ROI. To reveal the relationship between activity in those ROIs and performance under noise masking conditions, individuals' mean activities across −12 to 8 dB SNRs in each ROI and mean accuracies across syllables and SNRs (−12 to 8 dB) were subjected to a Pearson's correlation for each group separately. Multiple comparisons were corrected with a FDR *q*=0.05 using Benjamini–Hochberg^41^ procedure. For each ROI, the correlation coefficients from two groups were also converted into *z*-scores using Fisher's *r*-to-*z* transformation^42^ and compared using the formula^22^ as follows:


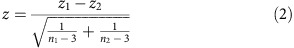


where *z*_1_ and *z*_2_ are the Fisher's *z*-scores of each group's correlation, *n* is the sample size of each group. This test gave a *z*-value that had a statistical signification indicating whether the difference between two correlation coefficients was significant.

### MVPA

Given the likelihood of high inter-subject anatomical variability and fine spatial scale of phoneme representations, we trained pattern classifiers to discriminate neural patterns associated with different phonemes and then tested these classifiers on independent test trials within anatomically defined ROIs. To do so, we first used the AFNI program 3dLSS (Least Square Sum regression^43^) to estimate univariate trial-wise β-coefficients for all brain voxels from the concatenated data.

We then used Freesurfer's (version 5.3 (ref. 44) automatic anatomical labelling (‘aparc2009' (ref. 45) algorithm to define a set of 148 cortical and subcortical ROIs using individual's high-resolution anatomical scan. For each noise level, MVPA was carried out within each anatomical ROI using shrinkage discriminant analysis^23^ as implemented in the R package ‘sda.' Shrinkage discriminant analysis is a form of linear discriminant analysis that estimates shrinkage parameters for the variance-covariance matrix of the data, making it suitable for high-dimensional classification problems. To evaluate classifier performance, we used five-fold cross-validation where each fold of data consisted of the β-regression weights of four of the five runs, with one run held out for testing. The shrinkage discriminant classifier produces both a categorical prediction (that is, the label of the test case) as well as a continuous probabilistic output (the posterior probability that the test case is of label x). The continuous outputs were used to compute the AUC metrics, and the AUC scores were used as an index of classification performance because they are robust to class imbalances and are better able to incorporate the relationship between probabilistic classifier output and discrete category membership. Because the experiment had four phoneme categories, we used a multiclass AUC measure that was computed as the average of all the pairwise two-class AUC scores.

We then limited the statistical analyses in ROIs known to be sensitive to tasks involving the production and perception of speech. We used Neurosynth^46^ to create a meta-analytic mask using the search term ‘speech.' This resulted in a coordinate-based activation mask constructed from 424 studies and encompassing the language-related areas in the temporal and frontal lobes. We intersected this meta-analytic mask with the Freesurfer aparc 2009 ROI mask as defined in MNI space. If any of the intersected ROIs had≥10 voxels, we included that ROI in our analyses. To ensure hemispheric symmetry, if a left hemisphere ROI was included so as its right hemisphere homologue. This resulted in an ROI mask consisting of 38 ROIs (19 left and 19 right, Fig. 3).

Because MVPA was performed in anatomically defined ROIs specific to each participant, no spatial normalization was applied. Since the AUC score did not differ with phonemes in selected ROIs (POp, HG, STG and PT in the left hemisphere, F_3,45_<2.46, *P*<0.075, repeated-measures ANOVAs), significance of classification at the group level in each of the 38 ROIs at each noise level was evaluated by a one-sample *t*-test on individuals' phoneme-averaged AUC scores, where the null hypothesis assumed a theoretical chance AUC of 0.5. The effect size was estimated using Cohen's d^47^. Multiple comparisons were corrected with a FDR *q*=0.05 using Benjamini–Hochberg^41^ procedure. The AUC scores were also subjected to a mixed ANOVA with ROI as the within-subject factor, group as the between-subject factor and SNR as the covariate to evaluate the group difference in classification. To display statistics at the group level, the statistic of interest was projected on the parcellated (aparc 2009 (ref. 45) cortical inflated map associated with the Freesurfer average template (‘fsaverage') using SUMA.

MVPA was performed within anatomical ROIs rather than a moving ‘searchlight'^48^ because we wished to preserve borders between spatially adjacent areas (for example, IFG and STG) that exhibited differential phoneme specificity at noisy conditions^14^. It would also improve classification sensitivity for certain regions (for example, STG) that showed distributed phonological representations^49^. For the left preCG, regional MVPA may not be optimal to disentangle speech- and response-related activities in articulatory and hand areas of left premotor/motor cortex, respectively. Although the classifiers were trained to discriminate speech-related rather than response-related activities, the classification may capture the button/finger decoding in addition to the phoneme category decoding in the left preCG. Indeed, the classification on responses using all the incorrect trials across SNRs was significant in the left preCG (*t*_15_=3.5, *P*=0.003, one-sample *t*-test, Supplementary Fig. 1), suggesting reliable button/finger decoding in the left preCG. Also, the classification performance on stimuli and/or responses using all the correct trials was higher than the classification on responses using all the incorrect trials in the left preCG, although the difference was not significant (*t*_15_=1.447, *P*=0.168, paired *t*-test). This supports the possibility of button/finger decoding component in stimulus-based classification in the left preCG. Note that we do not emphasize the classification performance in the left preCG in our study, and the contamination of button/finger decoding on phoneme classification was not found in other regions. For instance, in the hand-control area (right preCG), adjacent somatosensory cortex (left postCG) and four core regions of the sensorimotor integration model (left POp, HG, STG and PT), the classification on stimuli using correct trials was significant (all *t*_15_>3, *P*<0.01), but the classification on stimuli or responses using incorrect trials was not significant (all *t*_15_<1, *P*>0.1).

To reveal how sensorimotor integration as a function of noise differed with age, AUC scores in frontal POp and three auditory ROIs (HG, STG and PT) in the left hemisphere, core regions of the sensorimotor mapping model (Fig. 5a), were tested by mixed ANOVAs with ROI (2 levels: POp and one of the auditory ROIs) and SNR as the within-subject factors and group as the between-subject factor. AUC difference scores between pairwise ROIs were further subjected to mixed ANOVAs with SNR as the within-subject factor and group as the between-subject factor to evaluate the group difference in sensorimotor mapping function. This was followed by one-way (SNR) repeated-measures ANOVAs and one-sample *t*-tests to reveal the pattern of sensorimotor integration function for each group separately.

To determine whether differences in phoneme classification between regions or between age groups were related to differences in BOLD activity, the mean activities across syllables in two critical anatomical ROIs (left POp and left STG) were extracted for each noise level and each group. A mixed ANOVA with ROI and SNR as the within-subject factors and group as the between-subject factor was used to test the main effects and interactions.

Finally, the relationships between activity in the left POp spherical ROI (−50, 14, 18; 8-mm radius, defined as showing age-related upregulation of activity with age-equivalent performance), phoneme specificity in four core regions (the left POp, HG, STG and PT) and behavioural accuracy were investigated to unravel the nature of age-related frontal upregulation. For each group, individuals' mean AUC scores across −12 and 8 dB SNRs in each of the four ROIs were correlated with mean activities across those SNRs in the left POp spherical ROI and the mean behavioural accuracies across those SNRs by Pearson's correlations followed by FDR correction^41^. For each ROI, the correlation coefficients from two groups after Fisher's *r*-to-*z* transformation were also compared and corrected for FDR^41^.

### Data availability

Data that support the findings of this study are available from the corresponding author on request.

## Additional information

**How to cite this article:** Du, Y. *et al*. Increased activity in frontal motor cortex compensates impaired speech perception in older adults. *Nat. Commun.* 7:12241 doi: 10.1038/ncomms12241 (2016).

## Supplementary Material

Supplementary Information Supplementary Figure 1

## Figures and Tables

**Table 1:** Contrast of BOLD activity between young and older adults when both groups achieved equal accuracy (*P*FWE<0.01).

Brain regions	Brodmann's area	Peak Talairach coordinate			*t*-value	No. of voxels
		*x*	*y*	*z*		
*Older>young*						
L/R thalamus	NA	−19	−16	7	−5.75	392
L pre/postcentral gyrus	3, 4, 6	−43	−16	45	−5.25	476
L superior temporal gyrus/middle temporal gryus	21, 22	−51	−20	−6	−5.23	168
L superior parietal lobule	7	−30	−51	56	−4.78	152
L pars opercularis/ventral premotor cortex	44, 6	−50	14	18	−8.354	146
L medial frontal gyrus	6	−12	−20	50	−5.23	100
L middle frontal gyrus	10	−40	52	5	−5.05	89
L insula/inferior frontal gyrus	13, 47	−29	20	−4	−4.55	65
R pre/postcentral gryus	3, 4, 6	33	−21	45	−7.66	517
R superior temporal gyrus/middle temporal gyrus	21, 22	50	−14	−4	−6.03	269
R medial frontal gyrus	6	12	−18	50	−5.81	242
R superior parietal lobule	7	18	−62	56	−5.08	168
R precuneus	31	14	−56	22	−5.308	90
						
*Young>older*						
R inferior parietal lobule	40, 39	47	−53	49	5.20	99

Abbraviations: BOLD, blood oxygenation level-dependent; NA, not applicable; *P*FWE, family-wise error-corrected *P*-value.

**Table 2:** Regions with significant (FDR corrected *P*<0.05) phoneme classification at each SNR in each group.

SNR	Region-of-interest	AUC score	*t*-value	Uncorrected *P*-value	Cohen's d
*Young*					
No noise	L Heschl's gyrus	0.528	3.635	0.002	0.909
	L superior temporal gyrus	0.529	7.161	0.000	1.790
	L planum temporale	0.532	4.171	0.001	1.043
	L supramarginal gyrus	0.526	3.429	0.004	0.857
	L postcentral gyrus	0.552	3.4	0.004	0.850
	L central sulcus	0.555	3.28	0.005	0.820
	L precentral gyrus	0.541	3.264	0.005	0.816
	L inferior frontal–pars opercularis	0.538	6.347	0.000	1.587
	L inferior frontal–pars triangularis	0.542	3.738	0.002	0.935
	R Heschl's gyrus	0.528	3.418	0.004	0.855
	R superior temporal gyrus	0.525	3.942	0.001	0.985
	R posterior lateral fissure	0.521	2.899	0.011	0.725
	R supramarginal gyrus	0.536	3.783	0.002	0.946
	R postcentral gyrus	0.530	2.801	0.013	0.700
	R precentral gyrus	0.536	3.05	0.008	0.763
8 dB	L postcentral gyrus	0.552	3.879	0.001	0.970
	L precentral gyrus	0.537	3.534	0.003	0.883
	L inferior frontal–pars opercularis	0.535	3.839	0.002	0.960
−2 dB	L postcentral gyrus	0.553	3.819	0.002	0.955
	L inferior frontal–pars opercularis	0.526	3.982	0.001	0.996
					
*Older*					
No noise	L postcentral gyrus	0.546	3.535	0.003	0.884
	L precentral gyrus	0.531	3.690	0.002	0.923
	L inferior frontal–pars opercularis	0.538	6.084	0.000	1.521
8 dB	L postcentral gyrus	0.540	3.662	0.002	0.916
	L central sulcus	0.547	4.766	0.000	1.191
	L inferior frontal–pars opercularis	0.526	3.534	0.003	0.883

Abbraviations: AUC, area under the curve; FDR, false-discovery rate; SNR, signal-to-noise ratio.
