# Supplementary material for: Increased activity in frontal motor cortex compensates impaired speech perception in older adults
Source: Nat Commun. 2016 Aug 2;7:12241. doi: 10.1038/ncomms12241 (PMC4974649; doi:10.1038/ncomms12241)
Supplement: Supplementary Information — Supplementary Figure 1 [file ncomms12241-s1.pdf]

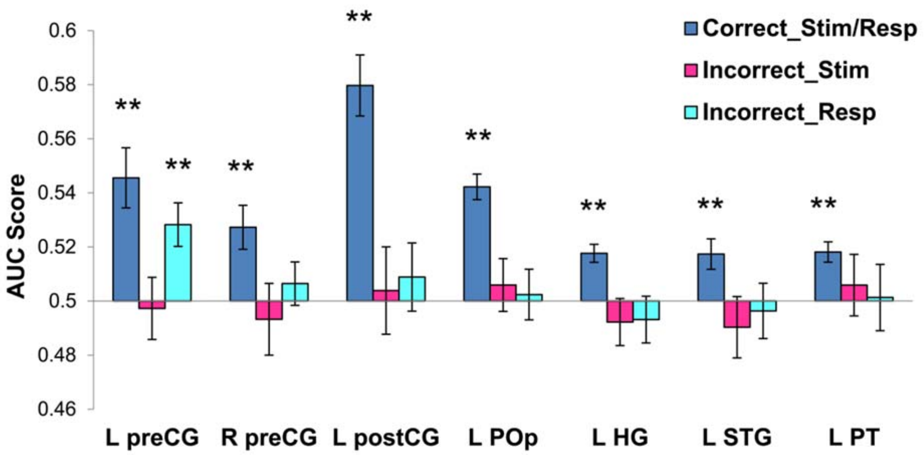

Supplementary Figure 1. Classification performance on stimulus or response patterns in young adults. Blue bars show classification performance on stimulus or response patterns using all the correct trials; red bars show classification performance on stimulus patterns using all the incorrect trials; green bars show classification performance on response patterns using all the incorrect trials. Note that the classifications based on stimuli or responses were the same for correct trials. ** $p < 0.01$ by one-sample t tests. HG, Heschl's gyrus; POp, pars opercularis; postCG, postcentral gyrus; preCG, precentral gyrus; PT, planum temporale; STG, superior temporal gyrus.
